# The perceptions of professional nurses regarding factors affecting the provision of quality health care services at selected rural public clinics in the Capricorn district, Limpopo Province

**DOI:** 10.4102/phcfm.v13i1.2830

**Published:** 2021-08-05

**Authors:** Nick T. Matlala, Rambelani N. Malema, Mamare A. Bopape, Peter M. Mphekgwana

**Affiliations:** 1 Department of Nursing Science, School of Health Care Sciences, University of Limpopo, Polokwane, South Africa; 2 Department of Research Administration and Development, University of Limpopo, Polokwane, South Africa

**Keywords:** quality, health care service, public clinic, primary health care clinic, primary health care, professional nurse

## Abstract

**Background:**

Despite many initiatives made by the National Department of Health through the Minister of Health, the provision of quality health care services remains a serious challenge in South Africa, especially in public rural clinics.

**Aim:**

The study aims to determine the perceptions of professional nurses on the factors affecting the provision of quality health care services at selected public primary health care clinics in rural areas of the Capricorn District, Limpopo Province.

**Setting:**

The study was conducted at selected public primary health care clinics in rural areas of the Capricorn District, Limpopo Province.

**Methods:**

This study utilised a quantitative method, descriptive and a cross-sectional study conducted for three months at the selected public primary health care clinics. A structured self-administered questionnaire was used to collect data from 155 professional nurses who met the selection criteria. Data were analysed using Statistical Package for Social Sciences programme version 26.0.

**Results:**

The results of 155 professional nurses were only 116 (74%) and reported that the use of modern technology such as electronic blood pressure, sonar machines and pulse reading computers improves the quality of health care services. Also 129 (84%), 124 (77%) and 118 (76%) reported that they were overwhelmed by the workload, the staff attitude and cleanliness of the clinic, respectively, which all affect the quality of health care services rendered. Moreover, only about 29 (19%) were satisfied with the salary they earned.

**Conclusion:**

Despite the effort and interventions put in place by the Department of Health with regard to the Ideal Clinic Realisation and Maintenance in response to the current deficiencies in the quality of primary health care services and to lay a strong foundation for the implementation of National Health Insurance. The quality of health care services is still hindered by several factors such as an overwhelming workload, the attitude of the staff and cleanliness in the work environment, poor infrastructure and the professional nurses perceive the environment as lacking equipment.

## Introduction

The quality of health care services refers to a degree that health services for individuals and populations increase the likelihood of desired health outcomes and are consistent with current professional knowledge.^1^ The provision of quality health care services globally remains the most important shared responsibility and opportunity to improve the health of the people.^1^ Professional nurses are the backbone of health care services. They are therefore legally and morally liable to provide quality health care services.^2^ However, the quality of health care services provided is determined by their ability to deliver and maintain standards which include reducing mortality, morbidity, ensuring availability, accessibility and affordability of health care. Furthermore, they should reduce waiting time, and ensure the safety and security of the patients.^2^

Primary health care (PHC) refers to the provision of integrated and accessible health care services by health care professionals who are accountable for addressing the large majority of health care needs.^3^ Primary health care is also an important nursing activity area, where nurses develop and articulate actions to promote, prevent and recover the population’s health.^3^ In the PHC setting, the working environment was perceived by professional nurses as not being suitable to the professional practice of nurses in rendering quality health care services.^4^ On the other hand, the nurses reported that in the working environment, they are exposed to different categories of occupational health risks and safety problems. Furthermore, these risks hinder the provision of quality health care services.^4^ In support of this, a study conducted in Iran on the factors influencing the quality of health care services found that the working environment was one of the factors that affect the quality of health care services rendered. The study also revealed that nurses expressed the need for a quiet and supportive physical environment to prevent the development of negative moods that may affect the quality of health services rendered to the patients.^5^

The African continent is developing, yet it is facing health care service challenges such as inadequate human resources and budget allocation to health care services. A study was conducted about identifying key challenges affecting the health care sector in Africa.^6^ The study found that poor leadership and management in health care, inadequate human resource and budget constraints in health care services were the three major problems which accounted to over two-thirds of the perceived problems in the health care sector in Africa.^6^ In sub-Saharan Africa, it was found that the shortage of human resources for health care was majorly blamed for the countries’ failure to provide quality health care services.^7^ Furthermore, only three to four doctors and 28.4 nurses per 10 000 people were available to render health care services which did not always translate to the provision of good health care services as many of the health facilities are severely short-staffed.^7^

In South Africa, in the post-apartheid era (1994), the following were the challenges that were facing the health care system in providing quality health care services: unequal distribution of resources such as budgetary allocations, inadequate human resources and slow progress in restructuring the health care services.^8^ It was further concluded that despite many initiatives made to improve the health care system services, millions of South Africans still suffer preventable harm.

The National Department of Health (NDoH) in South Africa flagged six areas that are fundamental to the provision of quality health care in all establishments. Six priority areas are positive, caring attitudes, the duration of waiting, cleanliness, patient safety, infection prevention and control, the availability of medicines and medical supplies.^9^ Furthermore, the NDoH established the National Health Insurance (NHI) plan, to ensure that everyone in the country has access to appropriate, efficient and quality health care services.^10^

The NDoH also designed a programme, the Ideal Clinic Realisation and Maintenance (ICRM) programme in response to the current deficiencies in the quality of PHC services. The ICRM programme was used to assess the clinics by focusing on the process to improve integrated clinical service management, infrastructure, human resources, waiting times, financial management and supply chain management.^11^ The number of clinics that scored over 70% increased from 139 to 445, whilst the clinics that scored below 40% dropped from 213 to 90. However, given that the country has 3477 PHC facilities, an achievement of only 322 ideal clinics leaves much to be desired.^11^

Despite many initiatives made by the NDoH through the Minister of Health, the provision of quality health care services remains a serious challenge in South Africa, especially in public rural clinics. Many PHC facilities in South Africa are still facing serious challenges such as long waiting duration and insufficient space to attend comfortably to service users. This has led to negative experiences of care, thus compromising the important role that PHC services play in some health promotion and disease prevention.

### Theoretical framework

**FIGURE 1:**
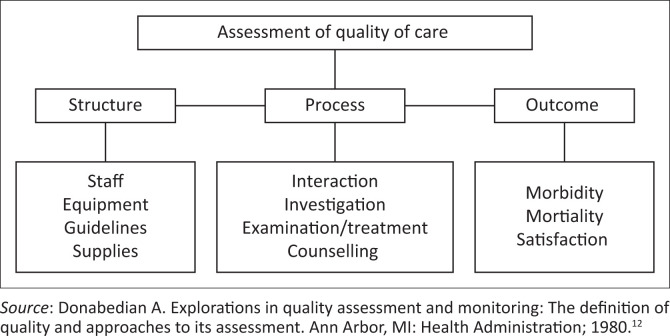
The Donabedian model for assessment of the quality of care.

Donabedian’s model was applied to guide this study (Figure 1). However, this model provides a framework for examining health care services and evaluating the quality of health care services. According to the model, information about the quality of health care can be divided into three domains, namely structure, the process and outcome.^12^

A structure refers to the required resources such as staff, equipment, infrastructure, policies and finances, which enables a health care provider to render quality health care services.^12^ In a study conducted on the factors influencing the quality of health care services in Iran, it has been reported that there is a shortage of equipment such as blood pressure machines, sonar machines, pulse oximeters, examination lamps, beds, haemoglobin and glucometer machines. Furthermore, the shortage of equipment increases job stress for the health care professionals which consequently affects the quality of their work.^5^

In 2012, the Ministers of Health and Finance were called to talk about the problem of poor infrastructure at clinics, which includes old buildings, inadequate consulting rooms and inconsistent power supply, especially in the rural areas.^13^ The health care services are essential and increasingly costly, and failure to provide free health care services is due to little to no effort is being performed towards public health care infrastructure. Therefore, the priority was to strengthen the capacity of public health care infrastructure to provide effective and safe health care services.

Process refers to the treatment process that includes interpersonal process factors and technical skills to render quality health care services.^12^ According to a report by the Department of Health in the United Kingdom, although health care professionals are experts in their fields, they may need further knowledge and technical skills to render quality health care services. Therefore, more acquired knowledge and technical skills will help to keep health care professionals up to date with what is changing in health care, such as new medications and more advanced equipment.^14^

Donabedian defined the outcome measure as a change in the patient current and future health status that can be attributed to receiving quality health care.^12^ The outcome measure remains the ultimate validator of the effectiveness and quality of health care to measure the effect of the services rendered by health care professionals to patients.^12^

These three domains, namely the structure, the process and outcome, are interdependent of one another, and they have been researched in this study.

## Methods

### Study design

The study followed a descriptive observational cross-sectional quantitative design to identify factors affecting the provision of quality health care services at public sector PHC clinics in three rural municipalities of the Capricorn District, Limpopo Province.

### Study setting

The study was conducted in the Capricorn District in Limpopo Province. The Capricorn District is located in Limpopo Province, which is one of the nine provinces in South Africa. The Capricorn District had five municipalities, namely Aganang, Blouberg, Lepelle-Nkumpi, Molemole and Polokwane. Thus, it had about five municipalities and three were selected, namely Blouberg, Lepelle-Nkumpi and Aganang, because they are predominantly rural, whereas Molemole and Polokwane municipalities were left out as they are urban.

According to Statistics South Africa Census 2011, Blouberg municipality has an estimated population of 176 135 people with a total of 43 747 households, and the majority of the population is black Africans who constitute 99% of the total population and live in the tribal areas.^15^ Aganang municipality is composed of 105 villages and has four traditional authorities, namely Moletsi, Matlala, Maraba and Mashashane. It is the fourth densely populated municipality and has a population of 131 164 and a total of 33 918 households. The municipality is dominated by black Africans who constitute 99.6% of the total population. Lepelle-Nkumpi municipality has an estimated population of 230 350 people with a total of 59 682 households. The municipality is the second largest in the district, harbouring 18% of the district population.^15^

The following health care services are rendered in the selected PHC clinics: reproductive health, maternal and child health care including family planning, immunisation, education concerning prevailing health problems and methods of preventing and controlling them, and appropriate treatment for communicable and chronic diseases and injuries, psychiatric conditions, sexually transmitted infections and the management of acute illness such as diarrhoea, pneumonia and promotion of nutrition.

### Study population

The Capricorn District had five municipalities, namely Aganang, Blouberg, Lepelle-Nkumpi, Molemole, and Polokwane. Of the five municipalities, three were purposively selected, namely Blouberg (19 clinics), Lepelle-Nkumpi (21 clinics), and Aganang (10 clinics) because they are predominantly rural, whereas Molemole and Polokwane municipalities were left out because they are regarded as urban. The three municipalities chosen had a total of 50 clinics. Twenty-five clinics were randomly selected from the 50 clinics by writing the names of all the clinics on pieces of paper and putting them in a container and randomly drawing 25 names. In the selected clinics, each PHC clinic had an average of seven professional nurses leading to a total of 175 professional nurses (725 = 175). The Census sampling method was used to select the professional nurses. This study included all professional nurses with two years of experience or more who were able to participate. Only 155 professional nurses eventually took part in the survey and 20 were excluded as they had less than two years of experience.

### Data collection

A previously validated questionnaire was adapted for use in the study.^16^ The questionnaire was intended to assess the factors affecting the provision of quality health care services in the public health sector in Kenya. For reliability, the test–retest method was used with Cronbach’s alpha of 0.83, suggesting that the study questionnaire tool was reliable and consistent. The researcher adapted the questionnaire with the study supervisor and after discussion with colleagues to evaluate the content in terms of whether it appeared to reflect the concepts the researcher intended to measure. The final questionnaire consisted of 32 questions that were divided into two sections:

- Section A: Demographic data (four questions) that included gender, educational level and work experience.
- Section B: Factors affecting the provision of quality health care services, which had 28 questions under the following headings: the number of patients seen per day, the impact of working conditions on health care service delivery, financial influence in the health care sector and the resources in health care.

A pilot study was conducted at Zebediela Gateway Clinic to check the appropriateness of the questionnaire and to identify unclearly formulated items. No changes were made to any of the questions as the questionnaire was found to be relevant to the objective of the study, free of errors and the respondents understood all the questions. The results of the pilot study were not included in the main study.

A letter requesting consent accompanied the questionnaire explaining the purpose of the study to the respondents. The respondents completed the questionnaires during lunchtime and it took 15 min – 20 min. The researcher was available during data collection to offer respondents clarity on questions they did not understand. Approximately, about two to three clinics were attended per day, depending on the distance between the clinics and the appointment days. The response rate was 100%, as all 155 questionnaires distributed were completed.

### Data analysis

A paper questionnaire was used to gather data from January 2018 to August 2018. The data cleaning and capturing was performed on Microsoft Excel and imported into Statistical Package for Social Sciences (SPSS) programme version 26.0 on a password-protected computer with the assistance of a university Bio-statistician. Frequencies and percentages were calculated to describe the perceptions of professional nurses on the factors affecting the provision of quality health care services.^17^

### Ethical consideration

Ethical clearance was obtained from the Turfloop Research Ethics Committee at the University of Limpopo (TREC/161/2016: PG). All professional nurses received oral and written information and gave informed consent in writing and verbally before they can participate in the study.

## Results

**FIGURE 2:**
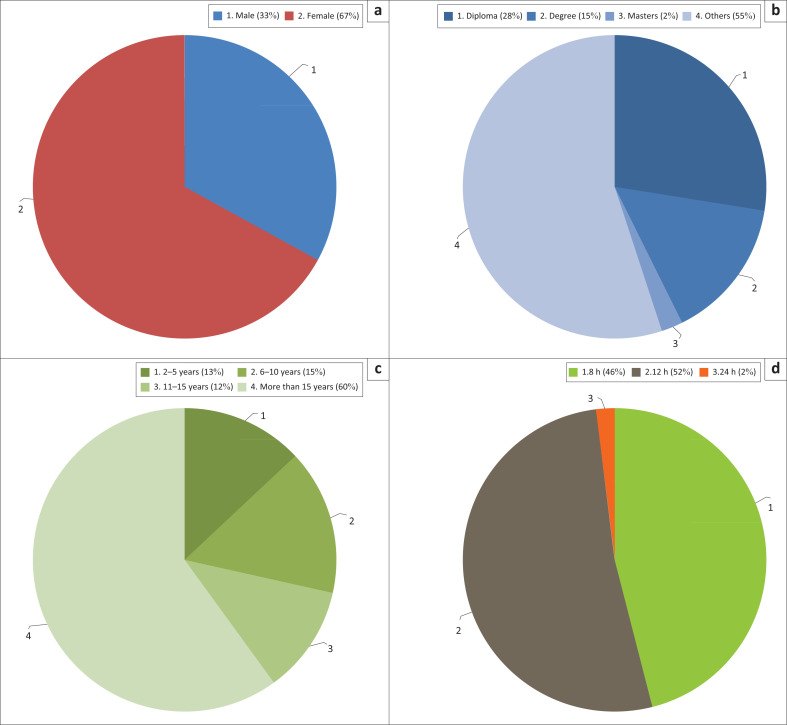
Demographic data: (a) gender, (b) education level, (c) work experience and (d) working hours.

In the study, female professional nurses represented the majority of the respondents, 102 (67%), compared to male professional nurses, 50 (33%), in public rural clinics of the Capricorn District with most respondents, 92 (60%), having a diploma in nursing as a qualification. Thirty-one professional nurses (31%) had 6–10 years of work experience. In the study, the majority of clinics were operating 12 h per day, that is, 80 (52%), followed by clinics operating 8 h per day, that is, 72 (46%), and clinics operating 24 h per day 2 (3%) (Figure 2).

Less than half of the professional nurses, 76 (49%), indicated that they have the required numbers of professional nurses with the necessary specialties to enhance the provision of quality health care services. Most participants reported that financial allocation by the government influences the quality of health care services provided in the clinic 109 (70%). Most professional nurses 102 (66%) reported that the government does not allocate enough funds for the smooth running of the clinics (Table 1).

**TABLE 1:** Structure.

Domains	Yes		No		Not sure	
	*n*	%	*n*	%	*n*	%
Do you have the required number of professionals with specialty to enhance the provision of quality health service?	75	80	63	41	14	9
Does the clinic has enough medical equipment to render quality health care services?	59	38	94	61	2	1
Does the use of available modern technology such as electronic blood pressure, sonar machines and pulse reading computers amongst staff improve service delivery to the public?	113	74	25	16	14	9
Is the maintenance plan of medical equipment regularly monitored?	31	20	116	75	8	5
Is the infrastructure of the clinic suitable to render quality health care services?	70	46	68	45	14	9
Are you involved in the planning of purchasing the necessary medical equipment?	79	51	74	48	2	1
Do you have enough consulting rooms at the clinic?	68	45	84	55	0	0
Is the clinic management invested in modern technology adequately in the institution to render health care services?	81	53	59	39	12	8
Does the government allocate enough funds for the smooth running of the clinic?	35	23	101	66	16	11
Does financial allocation influence the quality of health care services provided?	108	70	36	23	11	7
Are professional nurses free to voice their concerns about service delivery?	116	75	36	23	3	2

The findings of this study indicated that most professional nurses 116 (74%) reported that the use of modern technology such as electronic blood pressure, sonar machines and pulse reading computers improves the quality of health care services. Furthermore, (27%) of the professional nurses said that the high cost of modern technology affects the provision of quality health care services. Almost the same number of professional nurses, 71 (46%) and 70 (45%), respectively, said that the infrastructure of the clinic is suitable and is not suitable for the rendering of quality health care services (Table 1).

Most professional nurses, 116 (75%), reported that the maintenance plan of the medical equipment is not regularly monitored. Furthermore, 94 (61%) of the professional nurses reported that they do not have enough medical equipment in the clinics. Almost the same number of professional nurses, 71 (46%) and 70 (45%), respectively, indicated that the infrastructure of the clinic is suitable and not suitable to render quality health care services. The majority of the professional nurses, 116 (75%), said that staff members are free to voice their concerns about service delivery (Table 1).

The staff attitude was reported to affect the quality of health care services rendered as indicated by the majority of professional nurses, namely 121 (80%). Most professional nurses reported that patients are dissatisfied with the waiting time in clinics: 88 (57%). More than half of the professional nurses, 105 (68%), had attended in-service training in the past 6 months. The study found that the majority of the professional nurses, 129 (83.6%), were overwhelmed by the workload. Almost all the professional nurses, 149 (96%), indicated that health education was given daily. The majority of the professional nurses, 129 (83%), said that infection control is maintained in the clinics (Table 2).

**TABLE 2:** Process.

Domains	Yes		No		Not sure	
	*n*	%	*n*	%	*n*	%
Is the staff attitude good to render quality health care service?	121	80	23	15	8	5
Do the patients appreciate the waiting time they wait before service is rendered to them?	53	34	88	57	14	9
Have you attended in-service training in the past 6 months?	103	68	49	32	0	0
Do you believe other professional nurses render the quality of health care services to the patients?	126	81	11	7	18	12
Do professional nurses give health education to the patients daily?	149	96	4	3	2	1
Is infection control maintained at the clinic?	129	83	19	12	7	5
Is the mission and vision of the clinics stated?	144	93	5	3	6	4
Are you overwhelmed by the workload?	127	84	25	16	0	0

More than half of the professional nurses, 90 (58%), indicated that the number of patients seen daily at the clinic does affect the quality of health care services rendered to the patients. Furthermore, the majority of the professional nurses, 130 (84%), indicated absenteeism in the workplace to be affecting the rendering of quality health care services. Most professional nurses, 118 (75.7%), indicated that the cleanliness of the clinics affects the rendering of quality health care services. Less professional nurses, 31(20%), indicated to have sustained injuries on duty because of faulty medical equipment. Very few, 29 (19%), professional nurses were satisfied with the salary they earned (Table 3).

**TABLE 3:** Outcome.

Domains	Yes		No		Not sure	
	*n*	%	*n*	%	*n*	%
Does the workload of the clinic delay patients’ access to health care services?	118	58	29	34	8	8
Does absenteeism by other staff members affect the health care services rendered to the patients?	130	84	19	12	6	4
Does the cleanliness of the environment hamper the provision of health care services rendered in the institution?	115	76	29	19	7	5
Do you have injuries on duty by staff because of faulty medical equipment?	31	20	101	65	23	15
Are you satisfied with the way performance evaluations are performed?	70	46	73	48	9	6
Is salary compensation enough?	29	19	124	80	2	1

## Discussions

Discussions of the study are divided into three domains, namely structure, process and outcome as guided by Donabedian’s model.

### Structure

The study aimed to determine the perceptions of professional nurses on the factors affecting the provision of quality health care services at selected public PHC clinics in the rural areas of the Capricorn District, Limpopo Province. In this study, there were more female professional nurses than male professional nurses which concur with the findings of a study conducted in the North West province of South Africa, which reported that 92% of the professional nurses were women as compared to 7.6% men.^18^ The South African Nursing Council reported that when recruiting people into the nursing profession, there is no particular focus on gender because the responsibility of nurses is not gender-specific.^19^

The clinics that operate for 8 h daily were found to have a higher required number of professional nurses with specialty, to enhance the provision of quality health care services compared to clinics that are operating 12 h daily. As far as the nursing profession is dealing with patients’ life, it is of concern for professional nurses working for longer hours, that is, 12 h, as it affects the quality of work.^20^ Working long hours makes nurses less productive, distracts their attention and deteriorates physical and mental health which will further affect the quality of health care services rendered to the patients. Furthermore, long working hours will increase the risk of occupational injury as caused by fatigue, which will further affect their health and put the patients’ safety at risk and affect the provision of quality of health care services.^20^

The study by Donabedien indicated that equipment is one of the essential resources which enables health care professionals to render quality health care services.^12^ However, professional nurses in this study indicated that the clinics do not have enough medical equipment and consulting rooms to render quality health care services. This is similar to a study conducted on the barriers to quality patient care in rural health facilities in one region of Western Cape Province, South Africa^21^ where it was reported that the majority of the professional nurses indicated that they do not have enough equipment to render quality health care services.^21^ The World Health Organization (WHO) has shown that resources such as equipment are not allocated where they are needed most, that is, in rural poor health care sectors but are instead allocated to health facilities in large cities.^22^ Unless this is addressed, professional nurses are still going to struggle to render quality health care services because of a shortage of essential resources.

Most of the professional nurses reported that the use of modern technology such as electronic blood pressure machines, sonar machines and pulse reading computers improves the quality of health care services. Therefore, the expected outcomes in health care goals for the well-being of the patients will be beneficial to the patients. This includes outcomes such as moving to a paperless system that provides information at the right time (electronic medical records), moving towards bar-coded medications and automatic dispensing. This finding concurs with a study conducted in Kenya, which revealed that most of the health care professionals indicated that the use of modern technology improved health care services.^23^ In support, a report in America revealed that technology has changed and improved health care by providing new machines, medicines and treatments that save lives and improves the chance of recovery for billions of patients.^24^

In this study, almost the same number of professional nurses indicated that the infrastructure of the clinic is suitable and is not suitable for the rendering of quality health care services. This is consistent with a study conducted in Ghana which has reported that the health care providers,^25^ indicated that the clinic infrastructures are not suitable for the rendering of quality health care services. Furthermore, health care services in Ghana were often rendered in buildings and rooms with roof leakages.^25^ In South Africa, according to the Minister of Health in 2014, it was reported that the infrastructure in the poor public health care clinics is one of the main reasons why free quality health care fails.^13^ In this study, the model of Donabedian was used for examining the health care services and evaluating the quality of health care services.^12^ The structure which is one of the domains of the theory indicates that professional nurses view that many PHC facilities in rural areas still face serious challenges such as inadequate, old infrastructures and insufficient space to attend comfortably to service users.

### Process

The study found that the majority of the professional nurses were overwhelmed by the workload. In South Africa, there are 4200 public health facilities, and the number of people per clinic is 13 718, exceeding WHO guidelines of 10 000 per clinic.^26^ In the North West, the Department of Health reported that some clinics provide a 24-h service and approximately 300 patients visited the clinic per day. Overall, the clinic day shift consisted of five professional nurses, which resulted in one nurse attending to approximately 60 patients (1:60), thus exceeding the national norm of 1:40 patient to nurse ratio.^27^ A previous study by Tuten reported that professional nurses who are being overwhelmed by patient numbers can only endanger patients because of the mistakes that are more likely to happen once nurses have consulted beyond a certain number of patients.^28^

The majority of professional nurses have indicated that the staff’s attitude was reported to affect the quality of health care services rendered. The finding concurs with a study conducted in Kwazulu-Natal province that revealed that most of the nurses had a negative attitude towards patients. They verbally abuse patients, and in some cases, they neglected patients by withholding care.^29^ In support, the NDoH, the Minister of Health, has identified the staff’s attitude as a key health system challenge in the country and has therefore included it in the six ministerial priority areas for patient-centred care in striving towards the goal to achieve rendering of quality health care services.^9^

### Outcome

In this study, the professional nurses have identified cleanliness as one of the factors that affect the rendering of quality health care services in public clinics. This study ascertains baseline data on the thoroughness of cleaning from clinic to clinic to improve the environmental cleaning; and disinfection^30^ has shown that the environmental surfaces were assessed in the eight clinics and the overall rate of cleanliness ranged from 29% to 77% for examination rooms, common clinic areas and waiting rooms, respectively.^30^ This finding is of concern as the cleanliness of the clinics affects health care services because clinics play an important role in the transmission of potential health care pathogens and infections.^31^ The environment where health care services are rendered should be clean at all times to prevent cross-infection.^30,32^ Also, professional nurses indicated that patients may experience a decrease in self-worth and dignity in the health care sector, which lacks cleanliness.^32^

The majority of the professional nurses were not satisfied with the salary that they were paid. The findings of this study are similar to the study conducted by Tshitangano.^33^ Unsatisfactory salaries, as reported by nurses, contribute to most nursing personnel’s decision to resign or translate to private sectors which will lead to a shortage of staff on duty and hinder the provision of quality health care services to patients.^33^

### Limitations of the study

The selection of professional nurses from two districts in Limpopo Province, firstly, did not allow extrapolation of the results of the entire province. Secondly, the study was a cross-sectional design, and self-reporting data may be sources of potential biases. Thirdly, the study used a quantitative instrument to evaluate a largely qualitative topic. Despite these limitations, similar results from various previous studies tend to support the validity of our findings.

## Conclusion

Despite the effort and interventions put in place by the Department of Health with regard to the ICRM in response to the current deficiencies in the quality of PHC services, and to lay a strong foundation for the implementation of NHI. The quality of health care services is still hindered by several factors such as overwhelmed by the workload, the attitude of the staff and cleanliness in the work environment, poor infrastructure and lack of equipment as perceived by the professional nurses.
